# Multiplexed-Based Assessment of DNA Damage Response to Chemotherapies Using Cell Imaging Cytometry

**DOI:** 10.3390/ijms23105701

**Published:** 2022-05-20

**Authors:** Nadia Vezzio-Vié, Marie-Alice Kong-Hap, Eve Combès, Augusto Faria Andrade, Maguy Del Rio, Philippe Pasero, Charles Theillet, Céline Gongora, Philippe Pourquier

**Affiliations:** 1 Institut de Recherche en Cancérologie de Montpellier, INSERM U1194, Université de Montpellier, Institut Régional du Cancer de Montpellier, F-34298 Montpellier, France; nadia.vie@icm.unicancer.fr (N.V.-V.); marie.konghap@gmail.com (M.-A.K.-H.); eve.combes@inserm.fr (E.C.); augustofariaaf@gmail.com (A.F.A.); maguy.delrio@icm.unicancer.fr (M.D.R.); charles.theillet@inserm.fr (C.T.); 2 Institut de Génétique Humaine, CNRS, Université de Montpellier, Laboratoire Maintien de l’Intégrité du Génome au cours de la Réplication, F-34090 Montpellier, France; philippe.pasero@inserm.fr

**Keywords:** imaging cytometry, DNA damage response, DNA repair, biomarkers, anticancer drugs, oxaliplatin, ATR inhibitor

## Abstract

The current methods for measuring the DNA damage response (DDR) are relatively labor-intensive and usually based on Western blotting, flow cytometry, and/or confocal immunofluorescence analyses. They require many cells and are often limited to the assessment of a single or few proteins. Here, we used the Celigo^®^ image cytometer to evaluate the cell response to DNA-damaging agents based on a panel of biomarkers associated with the main DDR signaling pathways. We investigated the cytostatic or/and the cytotoxic effects of these drugs using simultaneous propidium iodide and calcein-AM staining. We also describe new dedicated multiplexed protocols to investigate the qualitative (phosphorylation) or the quantitative changes of eleven DDR markers (H2AX, DNA-PKcs, ATR, ATM, CHK1, CHK2, 53BP1, NBS1, RAD51, P53, P21). The results of our study clearly show the advantage of using this methodology because the multiplexed-based evaluation of these markers can be performed in a single experiment using the standard 384-well plate format. The analyses of multiple DDR markers together with the cell cycle status provide valuable insights into the mechanism of action of investigational drugs that induce DNA damage in a time- and cost-effective manner due to the low amounts of antibodies and reagents required.

## 1. Introduction

Despite the significant improvement that can be obtained with immune checkpoint inhibitors, conventional chemotherapies still occupy a major place in the drug armamentarium, and a large proportion of cancer patients remain confronted with treatment failures due to resistance mechanisms. This explains why there is still an active search for compounds with new mechanisms of action or for new synthetic lethal drug combinations that could be used as potential alternatives. The identification of a potential drug candidate that may enter clinical trials is a long and costly process that requires the validation of in vitro and in vivo studies using an increasing number of cellular and animal models to ensure its efficacy and the absence of toxicity and also to validate its cellular target(s). A key step that is inevitable in the early preclinical development of anticancer drugs is the evaluation of their activity on cancer cell proliferation. This is usually achieved using a wide range of cytotoxicity assays ranging from a simple cell count using a Malassez counting chamber or automatic cell counters [1] to more sophisticated colorimetric assays that indirectly measure the cell number by quantifying the total amount of proteins (e.g., Sulforhodamine B assay), or measuring the cell viability by quantifying the activity of specific metabolic enzymes (ex: MTT assay and its derivatives MTS, XTT, and WST, or resazurin reduction assays), or the ATP content (e.g., CellTiter-Glo^®^, ATPLite™) (reviewed in [2]). These assays are performed in 96-, 384-, or 1536-well plates and are particularly adapted to automation for the screening of large compound libraries; however, they do not give access to much information regarding the drug’s potential mechanism of action. Flow cytometry is another method that can also be used to evaluate the cell response to a cytotoxic drug because it can distinguish between live and dead cells with specific dyes that are excluded from viable cells while they penetrate into damaged cells. For instance, propidium iodide (PI) is extensively used to discriminate dead cells, which are permeable to this dye regardless of the mechanism of death, from live cells with intact membranes [3]. Although accurate, flow cytometry requires a relatively high number of cells, robust controls, and specific skills to operate and maintain instrumentation platforms on a routine basis [4]. Another important limitation of these methodologies is the absence of cell visualization, which may bias the result interpretations.

In recent decades, several companies have developed automated plate-based cell imaging cytometers such as the Celigo^®^ (Nexcelom Bioscience, Lawrence, MA, USA) [5], Opera (Perkin Elmer, Richmond, CA, USA) [6], IN Cell Analyzer 2200 (GE, Buckinghamshire, UK) [7], Spark^®^ Cyto (Tecan, Männedorf, Switzerland) [8], CELLAVISTA^®^ 4 and NYONE^®^ (Synentec, Elmshorn, Germany) [9,10], Cytation 5 (BioTek, Winooski, VT, USA) [11], SpectraMax MiniMax 300 (Molecular Devices, San Jose, CA, USA) [12], CellInsight CX series (Thermo Fisher Scientific Inc., Waltham, MA, USA) [13,14], or ImageXpress PICO (Molecular Devices, San Jose, CA, USA) [15]. These cytometers were developed to analyze cell survival by simultaneously evaluating live and dead cells and also various targets of interest within the same cells as long as these targets could be fluorescently stained. Therefore, they allow for a reduction in the number of experimental steps that could affect the assay robustness. These platforms are particularly adapted for the assessment of the phosphorylation status of key proteins that are involved in various signaling pathways, and that may play a role in cancer cell proliferation or cell response to anticancer drugs.

In this study, we used the Celigo^®^ platform to investigate the effects of a panel of chemotherapeutic agents on cell proliferation and cell death. This strategy allowed us to rapidly identify the cytotoxic or the cytostatic effect of the tested drugs and the contribution of each mechanism to cell growth inhibition. We also describe the experimental conditions to study the cell response to these chemotherapies that directly target DNA or that induce replicative stress, ultimately leading to lethal DNA double-strand breaks, by analyzing, on a multiplexed basis, the qualitative (phosphorylation status) and quantitative (number of labeled cells and their labelling intensity) changes in various DDR markers (H2AX, DNA-PKcs, ATR, ATM, CHK1, CHK2, 53BP1, NBS1, RAD51, P53, P21) within the same cells. We could observe that the activation of these DDR markers could be identified in cells depending on their cell cycle status, which is another advantage of this methodology. The results of our study clearly show a cell response heterogeneity to DNA damaging agents within the same population, which cannot be assessed by Western blotting. We could validate this methodology by testing the synergistic combination of oxaliplatin and the ATR inhibitor, VE-822, and confirm that VE-822 enhances replicative stress and increases lethal DNA double-strand breaks [16]. This study provides the first set of methodological protocols for the use of the Celigo^®^ image cytometer to analyze the mechanism(s) of DDR response to investigational drugs. They will serve as a basis for the development of new pertinent sets of markers involved in DNA damage signaling. 

## 2. Results

### 2.1. Dual Evaluation of the Cytotoxic and Cytostatic Effects of Antiproliferative Agents by Image Cytometry

The evaluation of the cytotoxic potential of any compound is usually the first step in the preclinical development of anticancer drugs. It is typically evaluated using high throughput screens in cancer cell lines where the drug’s effect on cell viability or cell death is measured. These in vitro assays are performed in multi-well plates in which a global quantification of living cells or dead cells in each well is obtained using colorimetric assays, fluorescence- or luminescence-based protocols. These assays give a relatively robust account of the drug activity, but they usually measure one single parameter. 

**Figure 1:**
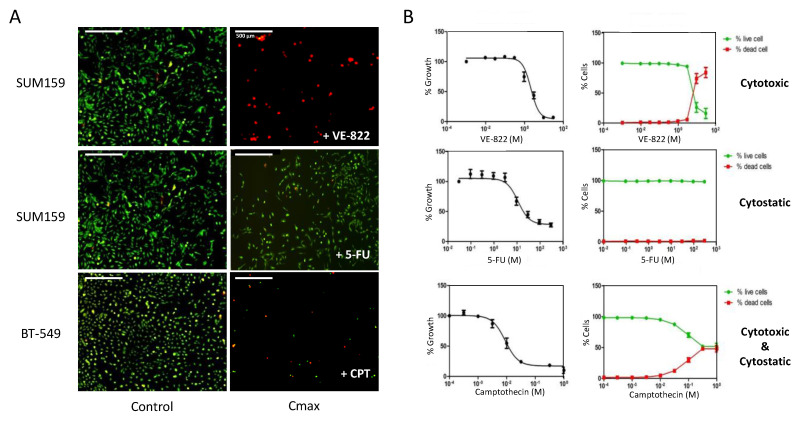
Dual assessment of the cytotoxic and the cytostatic effects of drugs using the Celigo^®^ imaging cytometer. (**A**) Triple-negative breast cancer cells (SUM159 and BT-549 lines), were incubated with increasing concentrations of each drug for 72 h. Then, dual staining with propidium iodide (PI) and calcein-AM was performed to visualize dead cells (red) and live cells (green), respectively, in each well. (**B**) Quantitation of the results obtained in panel (**A**) using the dedicated Celigo^®^ software. Right panels show the percentages of dead cells and live cells relative to untreated controls in function of the drug concentrations. Left panels: The total cell number (live + dead cells) in each well was calculated and normalized to untreated controls, and the percentages were plotted in function of the drug concentrations to obtain the IC_50_ values. Results are the mean ± SEM of ≥3 independent experiments.

Using image cytometry, we used established protocols to evaluate the effect of anticancer drugs on the cell number, cell viability, and cell death within the same population (Figure 1, Table 1). 

For this purpose, we seeded a panel of cancer cell lines of breast, prostate, and ovarian origin in 96-well plates, and we treated them with increasing concentrations of several cytotoxic agents that are known to induce DNA damage or replicative stress for 72 h. In most cases, we labelled cells with PI and calcein-AM without removing the supernatant to visualize dead and live cells, respectively, in each well prior to image acquisition (Figure 1A). Using dedicated software, we performed cell contouring based on each fluorescent label. When the cell shape and/or cell density precluded adequate contouring of calcein-AM-stained cells, we used nuclear staining with Hoechst. The representative images of PI (red) and Hoechst (blue) dual staining of OVSAHO and DU145 cells incubated with cisplatin and camptothecin, respectively, are shown as an example (Figure S1).

The image analyses allowed us to calculate the percentages of dead and live cells per well that were plotted as a function of the drug concentrations (Figure 1B, right panel). We also calculated the percentages of growth inhibition based on the total number of cells (live + dead) in each well, compared with untreated controls. We plotted these percentages as a function of the drug concentrations to calculate the IC_50_ values (drug concentration that inhibits 50% of cell growth) (Figure 1B, left panel). The results of Figure 1B illustrate the three main categories of effects that can be observed: drugs that are mainly cytotoxic, such as VE-822 in SUM159 cells; drugs that are primarily cytostatic, such as 5-FU in SUM159 cells; and drugs with both cytostatic and cytotoxic effects, such as camptothecin in BT-549 cells.

We also found that depending on the cell line, the growth inhibitory effect of a drug may not result from the same mechanism of action. Indeed, at the concentration of 0.1 µM, which inhibited the growth of all cell lines tested by ~80% (Figure S2A), camptothecin was mainly cytostatic in OVSAHO cells (less than 10% of dead cells). Conversely, it was primarily cytotoxic in HCC38 cells (80% of dead cells) and displayed both cytostatic and cytotoxic effects in DU145 and PC3 cells (Figure S2B).

Using image cytometry, we could rapidly obtain, in a single experiment, the IC_50_ values of several drugs as well as a quantitative assessment of the involved cytostatic and cytotoxic effects (Figure 1). Table 1 recapitulates data for nine different cancer cell lines and nine different drugs, leading to a color-coded classification that indicates the contribution of each type of mechanism. It is interesting to note that, regardless of the cell line, some drugs were mainly cytostatic, for instance, 5-FU, olaparib, and oxaliplatin to a lower extent. On the contrary, the decrease in cell number induced by camptothecin and VE-822 was mainly due to a cytotoxic effect. Our screen also revealed differential sensitivity to DNA damage depending on the cell line: breast cancer cell lines (especially HCC38 cells) were globally more sensitive to DNA damaging agents than prostate and ovarian cancer cell lines (Table 1). 

### 2.2. Image Cytometry for DDR Evaluation Using γH2AX as Marker 

**Figure 2:**
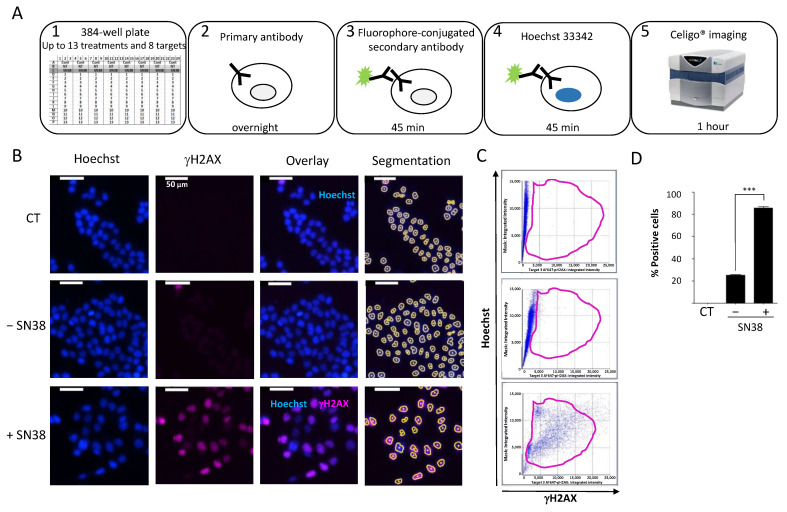
(**A**) Celigo^®^ imaging strategy used for DDR evaluation. (**B**) Representative fluorescence images of H2AX phosphorylation in HCT116 cells incubated (+) or not (−) with SN38 (2 µM, 24 h). Hoechst was used for nuclear staining and total cell count using the dedicated segmentation protocol (right panels). Images were acquired and analyzed using the Target + Mask application. CT: control cells stained only with the secondary antibody. (**C**) Representative flow-like analyses of the images obtained in (**B**). The dot plots show the Hoechst integrated intensity as a function of γH2AX integrated intensity. A gate (in purple) was defined for the control condition (CT) and applied to all other conditions to determine the percentage of positive cells and the fluorescence intensity in each cell. (**D**) Percentages of fluorescent positive cells for γH2AX labeling plotted in function of the cell treatment. The bar graphs represent the quantitative data (mean ± SD; *n* = 3) of the fluorescent images from one representative experiment; *** *p* < 0.0001 (Student’s *t*-test).

We then established a quantitative immunofluorescence assay to assess the DDR to commonly used anticancer agents. For this purpose, we used black-sided, flat-bottomed 384-well plates that allowed for the screening of the effects of two drugs at five concentrations on up to eight DDR markers in triplicate in a single run. We seeded cells at their optimal density and incubated them with the tested drugs 24 h later. We fixed and permeabilized cells directly in the well without trypsinization to preserve the phosphorylation status of the DDR markers. We then performed immunostaining with the specific antibodies according to the protocol outlined in Figure 2A.

To standardize each experiment, we used positive control cells incubated with a high concentration of the topoisomerase I inhibitor SN38 (2 µM) to set the dye exposures. To assess the background noise, we used untreated cells only stained with the secondary antibody (CT). First, we established the experimental settings to evaluate the DDR of HCT116 colon cancer cells to SN38 using γH2AX as a DDR marker (Figure 2B).

We concomitantly stained the cell nucleus with Hoechst to enable cell segmentation. We applied the obtained mask to each well to quantify each fluorescence signal in individual nuclei. We then analyzed representative images using the dedicated software. The analysis steps (Hoechst vs. γH2AX) were similar to those of standard flow cytometry analyses (Figure 2C). We then generated a gate based on the background staining (CT) and applied it to each condition. Using these settings, it is possible to calculate the number of fluorescent cells in each channel following drug treatment and also the mean integrated fluorescence intensity per cell. The results of our set-up experiment confirmed that SN38-mediated DNA damage induced H2AX phosphorylation, as evidenced by the significant increase in the percentage of fluorescent cells compared with controls (Figure 2D). These results further validated the use of image cytometry to rapidly and quantitatively assess the DDR to various antiproliferative agents in a multiplexed manner.

### 2.3. Evaluation of Other DDR Markers

We then extended our study to an additional panel of eleven DDR markers based on their reported key role in DNA damage signaling and/or repair: H2AX, DNA-PKcs, ATR, ATM, CHK1, CHK2, 53BP1, NBS1, RAD51, P53, and P21. Most of them are phosphorylated following DNA damage. 

**Figure 3:**
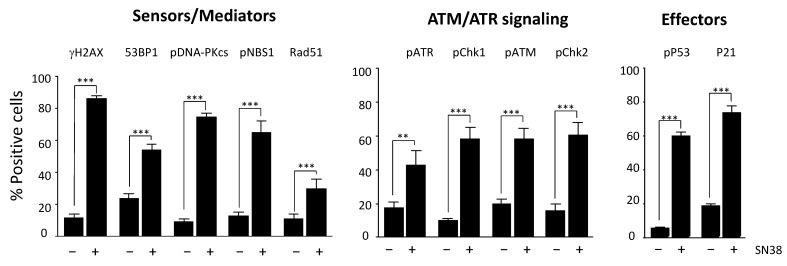
Monitoring of the HCT116 cell response to SN38 using a panel of DDR markers. Cells were incubated with 2 µM SN38 for 24h, and immunofluorescence staining was performed with antibodies against γH2AX, 53BP1, phosphorylated (p) NBS1, RAD51, pDNA-PKcs, pATR, pCHK1, pATM, pCHK2, pP53, and P21, as indicated in Materials and Methods. Fluorescent positive cells were quantified for each DDR marker in untreated cells and in cells incubated with SN38. Results are the mean ± SEM of ≥ 3 independent experiments; ** *p* < 0.01 and *** *p* < 0.001 (Student’s *t*-test).

We selected antibodies on the basis of their specificity and their suitability for immunofluorescence applications, as indicated in the manufacturers’ instructions (Table S1). We first identified the optimal experimental conditions for each DDR marker following incubation with SN38, particularly the dilution of each antibody (Figure S3). We then quantified DDR as described for γH2AX and determined the percentages of fluorescent-positive cells (Figure 3) for each marker in SN38-treated HCT116 cells. As expected, the results showed a significant increase in the number of fluorescent cells for all DDR markers analyzed, confirming that SN38 induces the activation of both the ATR/CHK1 and ATM/CHK2 pathways. Indeed, DNA topoisomerase I poisons are known to generate both single- and double-strand DNA breaks [17]. We also evidenced an increase in the number of cells with phosphorylated P53 (Ser 15) and P21, in line with what was reported previously [18]. In most cases, the increase in the percentage of fluorescent cells was accompanied by a significant increase in the mean fluorescence intensity (data not shown).

**Figure 4:**
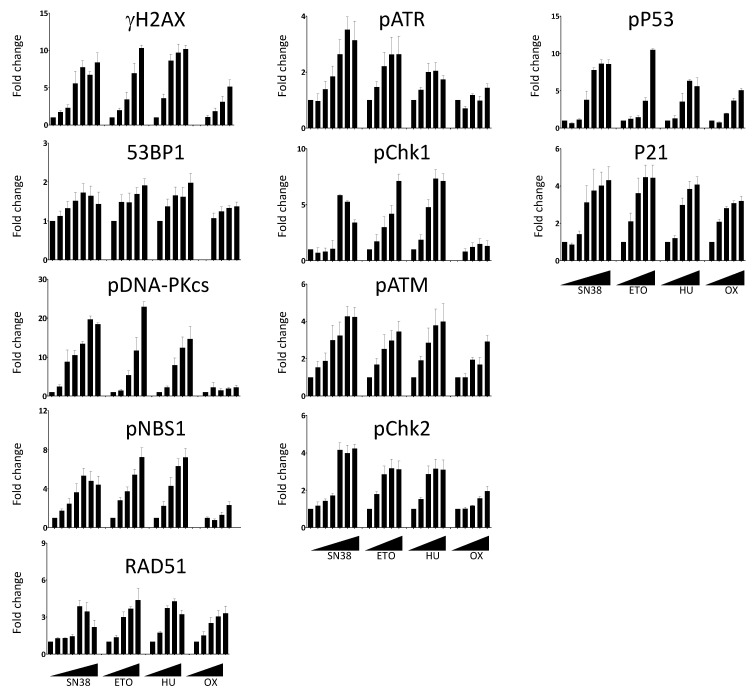
Dose-dependent DDR response of HCT116 cells to DNA damaging agents. Cells were incubated overnight with increasing concentrations of SN38 (0, 0.2, 1, 5, 16, 80, 2000 nM), etoposide (ETO; 0, 1, 2, 6, 30, 150 µM), hydroxyurea (HU; 0, 0.12, 0.6, 3, 15 mM), or oxaliplatin (OX; 0, 0.4, 2, 10, 50 µM), and DDR markers were evaluated as indicated in Figure 3. The results show the ratios of the mean number of fluorescent cells for each DDR marker following treatment compared with untreated cells. Results are the mean ± SEM of 2–4 independent experiments.

We then extended our analyses to compare the effects of SN38 and of three other DNA damaging agents with different mechanisms of action: etoposide, hydroxyurea, and oxaliplatin (Figure 4). Using the same experimental setup, we performed dose-response experiments and measured the changes in the phosphorylation of DDR markers. We then quantified the number of fluorescent-positive cells for each marker as previously described. We expressed the results as fold changes compared with untreated cells to allow the comparison among drugs because the basal fluorescence level and phosphorylation induction were different in function of the drug and the DDR marker. We observed an increase in the number of fluorescent cells in most cases, generally drug concentration-dependent (Figure 4). However, the extent of the increase was quite variable, depending on the marker and the drug used. For example, the number of cells with phosphorylated DNA-PKcs following incubation with SN38 or etoposide was increased by ~20-fold for concentrations inducing >75% of growth inhibition (Figure S4) whereas this increase was limited to ~2-fold upon incubation with oxaliplatin (Figure 4).

In the case of 53BP1, the increase in the number of fluorescent cells was marginal and never exceeded two-fold, regardless of the drug and of the concentrations used. We also evaluated the effects of the incubation duration on the DDR marker status by quantifying the percentages of γH2AX-, phosphorylated CHK1-, and phosphorylated CHK2-positive cells at the 5 h and 20 h time points (Figure S5).

As anticipated, the number of fluorescent-positive cells was generally lower when cells were incubated for 5 h than for 20 h. This was not the case for oxaliplatin-treated cells in which the increase in the percentage of γH2AX was more pronounced at 5 h than at 20 h. This suggested different kinetics of DNA damage signaling for this platinum derivative (Figure S5). This was also the case for hydroxyurea, an inhibitor of the ribonucleotide reductase that blocks DNA replication by depleting the pool of DNA precursors by inhibiting ribonucleotide reductase [19]. Hydroxyurea induces replication fork stalling and S-phase arrest that lead to DNA damage, including double-strand breaks [20]. We did not detect DNA damage signaling after a 5 h treatment. Conversely, H2AX phosphorylation was significantly enhanced following a 20 h treatment, suggesting that drugs acting on nucleotide synthesis may require longer incubation time for testing.

### 2.4. Cell Cycle-Specific Analysis of DDR Markers

**Figure 5:**
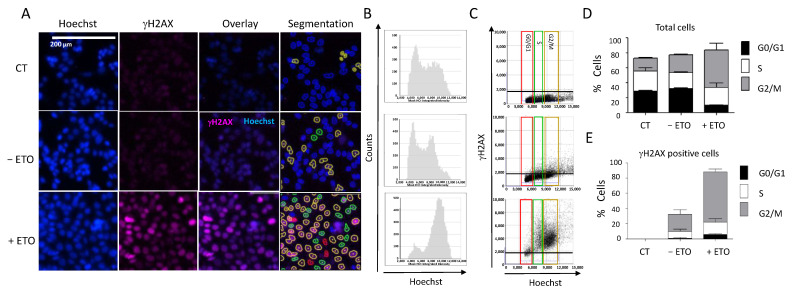
DDR response and cell cycle analyses. (**A**) HCT116 cells were incubated with 100 µM etoposide for 20 h. Cells were then fixed and stained for DNA with Hoechst and for γH2AX, and plates were analyzed using the Celigo^®^ system, as described in Figure 3. Representative fluorescence images are shown for each condition. Hoechst staining was used for segmentation and to quantify the DNA content in each cell. (**B**) Cell cycle profiles showing the total cell distribution in function of their DNA content, evaluated by the Celigo^®^ flow cytometry interface. (**C**) Distribution of γH2AX-positive cells in function of their cell cycle phase, determined by the segmentation process according to the following color code: red = G0/G1, green = S, brown = G2/M. (**D**,**E**) Quantitation of the results shown in (**B**,**C**), respectively. Three independent experiments were performed. Results are the mean ± SD of a technical triplicate from one representative experiment. CT: untreated cells stained only with the secondary antibody.

Another interesting feature of the Celigo^®^ image cytometer is the possibility to determine the cell cycle distribution concomitantly with the DDR marker status within the same cell population, thanks to the Hoechst staining used for both cell counts and DNA content determination (Figure 5). We incubated HCT116 cells with etoposide (100 µM, 20 h) followed by concomitant γH2AX and Hoechst staining, as previously described. We observed a significant increase in the number of fluorescent cells positive for γH2AX in etoposide-treated cells, which confirmed various studies (Figure 5A). We could visualize the integration intensity of the Hoechst as a histogram plot showing the cell cycle distribution within the cell population (Figure 5B).

Image analyses showed that etoposide treatment induced cell accumulation in the G2/M phase (Figure 5B,C): 66% of the treated cells in the G2/M phase compared with 22% of the untreated cells (Figure 5D). This was consistent with the results obtained when we quantified the percentages of H2AX-positive cells at each phase of the cell cycle (Figure 5E). Therefore, using image cytometry, it is possible to follow the change in DDR marker within each cell of the population and also determine the cell cycle phase in which such a change occurred, which provides precious indications on the mechanism of the tested drugs.

### 2.5. Multiplexed Analyses

**Figure 6:**
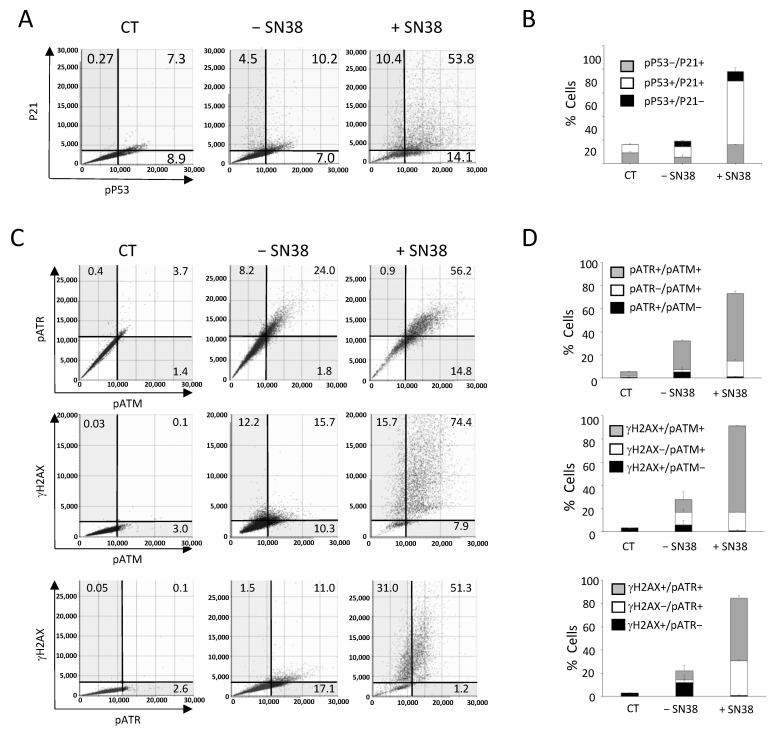
Multiplexed analyses of DDR markers following DNA damage. HCT116 cells were incubated with SN38 (2 µM) for 20 h. Then, multiplexed immunofluorescence labeling with anti-phosphorylated (p) ATM, -pATR, -γH2AX or -pP53 and -P21 antibodies was performed and analyzed with the Celigo^®^ flow cytometry interface using a gating that was determined for the CT condition (untreated cells stained only with the secondary antibody). (**A,C**) Representative dot plots show the distribution of fluorescent cells in function of their DDR marker status. (**B,D**) Quantitation of the results shown in (**A,C**). Independent experiments were performed twice for pATM/pATR/γH2AX labeling, and three times for P21/pP53 labeling. Results are the mean ± SD of a technical triplicate from one representative experiment.

The Celigo^®^ image cytometer can be equipped with four LED-based fluorescent channels allowing multiplexed analyses of up to three DDR markers together with Hoechst nuclear staining. For example, we could analyze HCT116 cells incubated with SN38 (2 µM, 24 h) by concomitant labeling for two downstream effectors of DNA damage signaling, phosphorylated P53 (pP53) and P21, and staining with Hoechst (Figure 6A). Before the experiment, we optimized the conditions for DDR marker multiplexed analysis for each marker individually to validate the absence of overlap of the fluorescence signals in each channel, as illustrated in the flow-like analyses of the obtained images (Figure S6A). Using these settings, the results showed that the percentages of pP53+, P21+, and pP53+/P21+ fluorescent cells were relatively low in untreated conditions (7%, 4.5%, and 10.2%, respectively) (Figure 6A). The incubation with SN38 increased the percentage of cells positive for a single marker by ~two-fold (from 7.0% to 14.1% for pP53 and from 4.5% to 10.4% for P21) and by ~five-fold the percentage of cells that were fluorescent for both pP53 and P21 (10.2% vs. 53.8%), which represented the majority of all fluorescent cells (Figure 6B and Figure S6B). Of note, we also observed a net increase in the mean signal intensity in pP53+/P21+ fluorescent cells (Figure 6A). These results confirmed our previous analyses using each marker individually (Figure 3). They also demonstrated the interest in using multiplexed labeling because it allows for the identification of cell populations with single- or double-fluorescent labeling, the percentage of which is specifically enhanced by the treatment. 

We then used our multiplexed analyses to concomitantly assess three DDR markers that are known as early response markers of DNA damage signaling: γH2AX, phosphorylated ATM (pATM), and phosphorylated ATR (pATR). We incubated HCT116 cells with SN38 (2 µM, 24 h), and staining was performed with the antibody pool and Hoechst according to experimental settings that were also validated by flow-like analyses of the obtained fluorescence images (Figure S7).

The results showed that in untreated cells (− SN38), the percentages of γH2AX-, pATR-, and pATM-positive cells for a single or for two markers reached ~25% (Figure 6C,D), in agreement with our previous analyses performed with each marker alone (Figure 3). The incubation with SN38 led to a global increase in the phosphorylation of the three DDR markers. Consequently, the percentage of fluorescent cells reached 90% for γH2AX, 80% for pATM, and 60% for pATR (Figure 6C,D). Our multiplexed analyses also showed that most cells in which ATM or ATR was phosphorylated were also positive for γH2AX. Indeed, the percentage of γH2AX+/pATR+ and γH2AX+/pATM+ cells increased by ~five-fold following SN38 treatment (11% vs. 51.3% and 15.7% vs. 74.4%, respectively) (Figure 6C,D). These data confirmed that HCT116 cell incubation with SN38 could produce DNA damage, as evidenced by the phosphorylation of both ATM and ATR, leading to H2AX phosphorylation and the subsequent activation of the ATM/CHK2 and ATR/CHK1 signaling pathways (Figure 3). These results further demonstrate that image cytometry can identify a subpopulation of cells in which DDR markers are concomitantly phosphorylated following incubation with the tested drug and can orient towards its mechanism of action and/or resistance.

### 2.6. Validation of the Multiplexed Approach Using the VE-822 + Oxaliplatin (VOX) Combination

Next, we used our multiplexed protocol to test the VOX combination in which platinum salt oxaliplatin and the ATR inhibitor VE-822 are combined. Using in vitro and in vivo colon cancer cell models, we previously demonstrated that ATR inhibition by VE-822 enhances oxaliplatin cytotoxicity and could potentiate the formation of DNA single- and double-strand breaks leading to apoptosis [16]. Using standard Western blotting, we previously showed that compared with untreated cells, VOX leads to enhanced phosphorylation of several DDR markers, including ATM (Ser 1981; by 6-fold), CHK2 (Thr68; by 12-fold), and P53 (Ser 15; by 20-fold) [16]. Conversely, phosphorylation of ATR (Thr1989) and CHK1 (Ser 345) was only slightly increased (1.2-fold and 1.9-fold, respectively) [16]. These DDR markers were activated (phosphorylated) by oxaliplatin alone; however, the addition of VE-822 only increased the phosphorylation of ATM, CHK2, and P53, indicating that the VOX synergistic effect is mainly associated with ATM/CHK2 pathway induction [16]. We confirmed these results by image cytometry analyses of each DDR marker individually (Figure S8).

**Figure 7:**
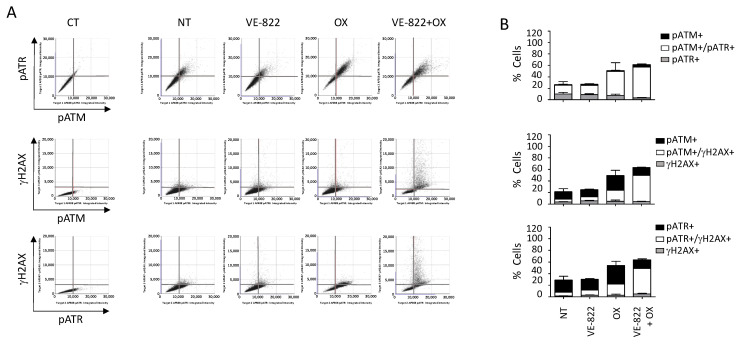
Multiplexed analyses of DDR markers following incubation with the VOX combination. HCT116 cells were incubated with VE-822 (1 µM) or/and oxaliplatin (OX; 2.5 µM) for 24 h, and DDR markers were analyzed as described in Figure 6. (**A**) Representative dot plots showing the distribution of fluorescent cells in the function of their DDR marker status. (**B**) Quantification of positive cells for each DDR marker in untreated cells and cells treated with VE-822 or/and oxaliplatin. Three independent experiments were performed. Results are the mean ± SD of a technical triplicate from one representative experiment.

**Figure 8:**
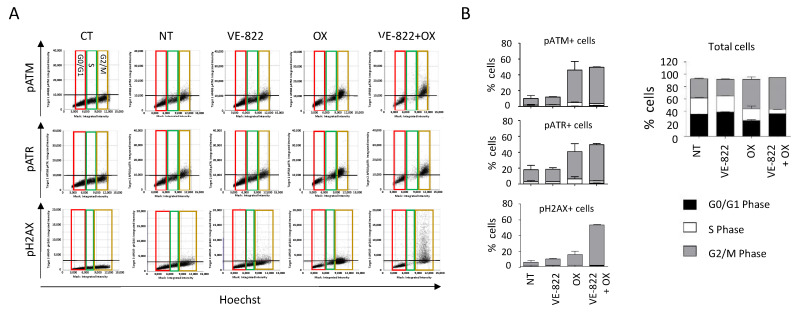
DDR marker analyses in the function of the cell cycle status following incubation with the VOX combination. HCT116 cells were incubated as in Figure 7, and the cell distribution in function of their DNA content and DDR marker was obtained using the Celigo^®^ flow cytometry interface. (**A**) Representative dot plots obtained for each DDR marker. Colored boxes indicate each phase of the cell cycle: red = G0/G1, green = S, brown = G2/M. (**B**) Distribution of total cells and of cells positive for each DDR marker in the function of their cell cycle phase. Three independent experiments were performed. Results are the mean ± SD of a technical triplicate from one representative experiment.

We also performed multiplexed analyses using the antibody combination against γH2AX/pATM/pATR according to the experimental conditions described earlier (Figure 7 and Figure 8). The percentage of cells positive for γH2AX, pATM, and pATR was increased following incubation with oxaliplatin compared with untreated cells: 19% vs. 1.9%, 46% vs. 10%, and 55% vs. 26%, respectively (Figure 7B and Figure 8B). As expected, the addition of VE-822 to oxaliplatin led to a drastic increase in the percentage of γH2AX-fluorescent cells to 55%, in accordance with the synergistic effect of the combination (Figure 7B and Figure 8B). Conversely, VOX did not significantly change the percentages of cells labeled for pATM and pATR. Our multiplexed analyses also revealed that most HCT116 cells positive for pATR and pATM were also positive for γH2AX (Figure 7). 

When cells were incubated with VOX, the cell-cycle analyses revealed a nearly complete abrogation of the S phase and the accumulation of pATM-, pATR-, and γH2AX-positive cells in the G2/M phase of the cell cycle (Figure 8). These results also revealed that compared with untreated cells, ~40% of the cells did not show any fluorescent staining following incubation with oxaliplatin or VOX (Figure 7B and Figure 8B). This indicates a heterogenous cell response to these drugs that could not be observed by standard Western blotting.

Together, these results confirmed the synergistic effect of VOX that was previously observed in oxaliplatin-resistant HCT116-R1 cells [16]. VOX induced an almost complete S-phase abrogation and the accumulation of cells in G2/M, which is consistent with the ATR role in the S and G2/M checkpoint and the repair of DNA breaks in G2 by homologous recombination [21].

Thus, using the Celigo^®^ image cytometer, we could set up adapted protocols to study in a short time frame the DDR to a series of anticancer drugs using a panel of key DDR markers. We highlighted the possibility of monitoring the activation of these markers in each phase of the cell cycle, providing additional insights into the mechanism of action of the tested drugs. Our results also emphasized the advantage of this multiplexed approach in terms of a lower number of cells and lower quantities of antibodies and reagents required, leading to important cost and time saving.

## 3. Discussion

The initial step in the preclinical development of anticancer drugs is the evaluation of their antiproliferative activity. This is usually achieved using a wide range of cytotoxicity assays that can be performed in 96-, 384-, or even 1536-well plates in a fully automated way, allowing the screening of large libraries of more than one hundred thousand compounds. These campaigns have led to the identification of many hits, as in the case of the public NCI60 anticancer drug screen program using a panel of 60 cancer cell lines [22]. However, these studies were limited by the lack of information regarding the potential mechanism of action of the identified hits. Flow cytometry-based assays can be used to assess the effects of drugs on cell viability or growth and on the activation (phosphorylation) of potential targets; however, one major limitation is the absence of cell visualization, which may limit the result interpretation, particularly the lack of data on morphologic cell features or subcellular localization of fluorescent markers [4]. 

Here, we used image cytometry as an alternative to overcome this limitation. We chose the Celigo^®^ image cytometer, which is particularly suitable for high-throughput screens because it accepts standard multi-well plate formats. It is equipped with an f-theta lens coupled with a galvanometer to provide a flat field that allows the analyses of the whole well surface [23]. With its four LED-based fluorescent channels, it is also well suited to multiplex labeling because it can assess up to three markers together with nuclear or cytoplasm dyes used for cell segmentation. We confirmed the advantages of this platform for the high-throughput evaluation of the effect of drugs on cell viability, using two dye combinations that could discriminate between dead and live cells, as previously reported [24]. However, we found that cytoplasmic calcein-AM staining could not be used for adherent cells with heterogeneous shapes or sizes and for cells growing in clusters (e.g., OVSAHO and DU145 cells) because these characteristics strongly affect the automatic segmentation process [25]. As an alternative, we used the Hoechst nuclear-specific dye that allowed for the monitoring of all of the cells in each well. We determined the effects of nine anticancer drugs on the viability of nine cancer cell lines. This allowed for the definition of three main categories of drugs depending on whether their growth inhibition was due to a cytostatic effect, a cytotoxic effect, or both. Increasing the drug concentration was often correlated with increased cytotoxicity; however, some drugs, such as 5-FU, which exert their activity through the inhibition of thymidylate synthase and the incorporation of metabolites into RNA and DNA [26], had a systematic cytostatic effect. Conversely, camptothecin, cisplatin, etoposide, and VE-822 showed a more pronounced cytotoxic effect across our cell panel because they induced ≥ 50% cell death at concentrations that were close to the IC_50_ values. Our results also showed that the balance between the cytostatic and cytotoxic effects of the drugs varied in the function of the cell model. This suggests that different signaling pathways of drug-induced DNA damage were triggered, probably due to the heterogeneity of the genomic background of these models, especially concerning their DDR gene status. Table 1 provides this information and also the IC_50_ values of the nine drugs tested in this study. These drugs, with known targets and mechanisms of action, could be used as a reference for comparison with investigational drugs. Interestingly, using this approach, Kuksin et al. demonstrated the similar efficiency of image cytometry and flow cytometry, with the advantage that the former allows excluding cellular debris that is often difficult to eliminate from gated cell populations [4].

Investigating the effects of DNA damage induced by genotoxic drugs is also crucial for the development of new potential anticancer agents. In this line, the detection and quantification of DNA double-strand breaks are of particular importance because they are usually associated with the lethal effect of these molecules [27,28,29,30,31]. Numerous studies have proposed experimental protocols using single-cell image analyses with fluorescence microscopy to quantify the DNA damage using the γH2AX [32], RPA/Rad51 [33], or NBs1/Rad51 [34] foci as biomarkers. This methodology remains the most sensitive for the quantitative and qualitative assessment of these foci but requires the use of glass coverslips and high-resolution microscopy (currently only semi-automated). Flow cytometry has been used as an alternative for high-throughput analyses, usually with the detection and the quantification of a single marker (e.g., γH2AX foci to directly assess DNA double-strand breaks [32], or RPA foci to quantify DNA end resection and DNA repair by homologous recombination [35]). To facilitate the detection of these markers bound to chromatin, an extraction step is required to remove unbound proteins [36]. Although reliable, flow cytometry requires a large number of cells that must be detached from their support before the analysis. Moreover, specific training is needed to routinely operate these platforms, and usually, the maintenance costs are high.

In this study, we showed that image cytometry is adapted to analyze, qualitatively and quantitatively, and on a high-throughput scale, the changes in the phosphorylation status of many DDR markers associated with DNA damage sensing and signaling. A previous study in which γH2AX fluorescence intensity was measured using image cytometry demonstrated that it is quite effective compared with other methodologies in terms of sensitivity, time, and cost per sample, while the cell number needed was minimal [37]. Though H2AX phosphorylation and the activation of other markers such as 53BP1 are evidenced by the formation of foci, we could not easily quantify these foci using this platform, as the magnification and resolution are not adapted to this kind of assessment, conversely to fluorescence microscopy. Nevertheless, an increase in the number of foci can indirectly be assessed by the global increase in average intensity, and we think that such a limitation will be overcome with next-generation devices. Here, we present dedicated protocols to assess DNA damage with an extended panel of eleven DDR markers (γH2AX, DNA-PKcs, ATR, ATM, CHK1, CHK2, 53BP1, NBS1, RAD51, P53, and P21), among which some can be evaluated concomitantly (multiplexed analysis). Our results clearly show the advantage of image cytometry for the rapid analysis of DDR signaling pathways at different levels in a single experiment, as exemplified by the re-assessment of the VOX combination (VE-822 and oxaliplatin). They also highlight the advantages of this technology. Indeed, very low amounts of antibodies were needed for the detection of each marker, thus drastically reducing the running costs compared with conventional Western blot analyses. Moreover, image acquisition of samples in a 384-well plate can be performed in less than 1 h, which is compatible with high-throughput screens. For instance, we could compare the effects of two drugs used at five concentrations in triplicate on the eleven individual markers in a single run using only two 384-well plates. Analyzing such a number of markers could also be useful to identify those that are activated by a given drug and rapidly test its combination with specific inhibitors of these markers to obtain synergistic effects. Furthermore, such analyses required a reduced number of cells per condition compared with flow cytometry, an interesting feature when slow-growing cell models (e.g., primary cells) are used. Nevertheless, one should anticipate the need for a large computational storage capacity due to the number of images generated per experiment. As image cytometry offers a visual analysis of the cells, it is also possible to discriminate between a global increase in fluorescence in the whole cell population and a high increase in fluorescence in a small subset of cells, which is impossible to assess using Western blotting. Another advantage of this platform is the possibility to perform multiplexed analyses using up to three DDR markers and nuclear staining for cell segmentation. The concomitant analysis of these markers and of the cell cycle status in each cell could also give insights into the heterogeneity of the cell response. Indeed, cell treatment with a given drug may activate all the markers of the same pathway in some cells but not in others in which it may not be fully operational, suggesting the existence of potential resistant clones. While this situation can be easily evidenced by multiplexed analyses of these markers using image cytometry, it cannot be assessed using Western blotting. The example of SN38 in our study is particularly telling as the treatment with this topoisomerase I inhibitor induced the phosphorylation of both ATM and ATR in only 62% of the HCT116 cells. Heterogeneity in the DDR response to other drugs used in this study has also been observed (data not shown). These results could be explained by a sensitivity problem of the method or a gating issue. However, it is most likely that cell-population heterogeneity in terms of cell cycle status or genetic background might translate into a heterogenous DDR, as previously reported [38]. 

Our study used traditional 2D cell culture systems for assessing the DDR in relation to anticancer drugs. However, image cytometry could be adapted to other cell culture systems. Cribbes et al. used a 3D model (spheroids) and glioblastoma U87MG cells to study the effects of fourteen anticancer drugs in 384-well plates [5]. They determined the spheroid size, invasion area, calcein-AM, PI, Hoechst, and caspase 3/7 fluorescence staining to evaluate cell viability and to generate a score that allowed for the classification of the drugs in terms of the function of their cytostatic or cytotoxic activity [5]. More recently, Mukundan et al. developed a spheroid image cytometry assay using the T47D breast cancer cell line and showed a concentration-dependent reduction in spheroids following incubation with six different anticancer drugs. This effect was correlated with the cell viability, measured by calcein-AM and PI fluorescence staining [39]. These studies demonstrate that image cytometry could be extended to more clinically relevant models, such as spheroids, organoids, and tissue samples, for high-throughput analyses. More studies are needed to determine whether DDR could be evaluated in these models by multiplexed analysis using our panel of markers.

## 4. Materials and Methods

### 4.1. Cell Culture 

Human cancer cell lines of breast (BT-549, SUM159, HCC38, MDA-MB-436), ovarian (OVSAHO and OVCAR-8), prostate (DU 145, PC-3, and 22Rv1), and colon (HCT116) origin were obtained from the TumoroteK bank (SIRIC Montpellier Cancer) and were authenticated by STR (short-tandem repeat) profiling. The cells were cultured in Dulbecco Modified Eagle Medium (BT-549, MDA-MB-436, DU 145) or RPMI 1640 (HCC38, PC-3, 22Rv1, HCT116) supplemented with 10% fetal bovine serum at 37°C in a 5% CO_2_ humidified atmosphere. The SUM159 cells were grown in Ham’s F-12 medium supplemented with 5% fetal bovine serum, 10 μg/mL of insulin, and 1 μg/mL of hydrocortisone. The cells were routinely tested for mycoplasma contamination using the MycoAlert^TM^ detection kit (Lonza, Basel, Switzerland).

### 4.2. Drugs and Reagents

Camptothecin, cisplatin, etoposide, 5-fluorouracile, gemcitabine, PF477736, hydroxyurea (HU), oxaliplatin, calcein-AM, bovine serum albumin (BSA), paraformaldehyde (PFA), Triton-X100, Hoechst 33342, propidium iodide (PI), and benzonase and dimethyl sulfoxide (DMSO) were purchased from SIGMA (Saint Quentin Fallavier, France). Olaparib, VE-822, and SN38 were purchased from Selleckchem (Euromedex, Souffelweyersheim, France).

### 4.3. Antibodies

Table S1 lists the antibodies used in this study and the corresponding working dilutions. Alexa Fluor 568-conjugated goat anti-rabbit and anti-mouse highly cross-adsorbed secondary antibodies (references A-11036 and A-11031) were purchased from Invitrogen (Thermo Fisher Scientific, Courtaboeuf, France).

### 4.4. In Vitro Cytotoxicity Assays

The effects of drugs on cell growth and cell death were measured simultaneously using the Celigo^®^ imaging cytometer as previously described [40]. Briefly, the cells were plated in black flat-bottom 96-well plates (Greiner, Courtaboeuf, France). After 24 to 48 h, exponentially growing cells were incubated with serial dilutions of the different drugs (added in triplicates to the cell medium) for 72 h. Then, the cells were stained by adding a mix of PI and calcein-AM (final concentrations of 1 µg/mL and 0.5 µM, respectively). Images were then acquired with the Celigo^®^ imaging cytometer, and PI-positive dead cells and calcein-positive live cells were quantified in each well using the dedicated software. Based on the total number of cells (live + dead), the growth percentage was calculated for each condition, compared with untreated controls, and plotted as a function of the drug concentrations to calculate the IC_50_ values (drug concentration required to inhibit the growth of 50% of cells). The results were the mean ± SEM of ≥3 independent experiments.

When the cell shape or cell density precluded adequate cell contouring following calcein-AM staining, dual staining with PI and Hoechst 33342 (final concentrations of 1 and 5 µg/mL, respectively) at 37 °C for 30 min was used. The number of live cells was then calculated by subtracting the number of dead cells from the number of total cells that were evaluated by Hoechst staining. 

### 4.5. Evaluation of the DNA Damage Response by Immunofluorescence

HCT-116 cells (1200 cells/well) were plated in black-sided, flat-bottomed 384-well plates (Greiner, Austria). The day after, cells were incubated with serial dilutions of the tested drugs for 20 h and were fixed in 4% PFA/0.1% Triton X-100 at room temperature for 10 min. After blocking with PBS/3% BSA for 1 h, primary antibodies diluted in PBS/1% BSA were added at 4 °C with gentle shaking overnight. For multiplexed assays, 2 or 3 different antibodies could be mixed. Then, the cells were washed with PBS/0.2% Tween 20 three times (5, 10, and 15 min) under shaking. Secondary anti-rabbit Alexa Fluor 568 (Invitrogen) or anti-mouse Alexa Fluor 488 antibodies were diluted in PBS/1% BSA (1/1000 and 1/500, respectively) as a mixture and incubated at room temperature with gentle shaking in the dark for 45 min. The γH2AX antibody, which is directly conjugated to the fluorochrome, was added for 45 min (at room temperature in the dark) after the secondary antibodies following three washes with PBS/0.2% Tween 20. After these steps, the cells were washed three more times and were kept in the dark, and incubated with 5 µg/mL of Hoechst 33342 at 37 °C for 30 min. The wells were then washed and left in PBS for analysis with the Celigo^®^ Cytometer imaging system.

### 4.6. Statistical Analysis

The Student’s *t*-test was used in GraphPad Prism (version 8.1.1) and the differences were considered statistically significant when *p* < 0.01 (**) and *p* < 0.001 (***).

## 5. Conclusions

The results of our methodological study confirm the interest in image cytometry to determine, at high throughput, the effects of a panel of drugs with different mechanisms of action on cell viability and cell death in adherent cancer cell lines of various origins. Our study also provides, for the first time, valuable methodological guidance for the evaluation of an extended panel of eleven DDR markers to analyze, on a multiplexed-basis, the DDR to various anticancer drugs using image cytometry. Although it is not intended to replace fluorescent microscopy or flow cytometry, our results highlight the time- and cost-effectiveness of image cytometry compared to other fluorescence-based methodologies as well as the low cell numbers required for data acquisition. 

## Figures and Tables

**Table 1:** Effects of DNA damaging agents on growth inhibition in various human cancer cell lines. Cells were incubated with increasing concentrations of each drug for 72 h continuously and processed as in Figure 1. Each value corresponds to the IC_50_ concentration (in µM) (concentration inhibiting cell growth by 50%) ± SD of 3 independent experiments. The color code corresponds to the effects (cytostatic or cytotoxic) of each compound that contribute to growth inhibition; green: primarily cytostatic, yellow: cytostatic ≥ cytotoxic, orange: cytostatic ≤ cytotoxic, red: mainly cytotoxic. Drugs’ mechanism of action: camptothecin: DNA topoisomerase I inhibitor; etoposide: DNA topoisomerase II inhibitor; cisplatin and oxaliplatin: DNA crosslinking agents; gemcitabine: DNA chain terminator; 5-FU: antimetabolite; Olaparib: PARP inhibitor; PF477736: Chk1 inhibitor; VE822: ATR inhibitor.

	Breast				Ovarian		Prostate		
	BT-549	SUM159	HCC38	MDA-MB-436	OVSAHO	OVCAR-8	DU 145	PC-3	22Rv1
**Camptothecin**	0.012 ± 0.005	0.008 ± 0.002	0.0078 ± 0.0037	0.01 ± 0.003	0.0093 ± 0.0006	0.0047 ± 0.0006	0.013 ± 0.003	0.012 ± 0.002	0.0185 ± 0.006
**Cisplatin**	1.33 ± 0.58	3.5 ± 2.5	4 ± 2.4	0.47 ± 0.16	2.75 ± 1.26	1.24 ± 0.31	1.6 ± 0.26	1.17 ± 0.40	2.43 ± 0.51
**Etoposide**	1.35 ± 1.05	0.37 ± 0.06	0.48 ± 0.22	0.16 ± 0.03	1.03 ± 0.45	0.46 ± 0.21	0.155 ± 0.013	n.d.	0.25 ± 0.06
**5-FU**	> 100	33 ± 12.0	> 100	9 ± 1	83.3 ± 28.8	8.33 ± 1.53	6 ± 3	13 ± 8.9	16 ± 2
**Gemcitabine**	0.007 ± 0.001	0.008 ± 0.004	0.0065 ± 0.003	0.006 ± 0.002	0.0087 ± 0.0011	0.004 ± 0.001	0.00325 ± 0.0006	0.008 ± 0.04	0.008 ± 0.0006
**Olaparib**	> 100	22.8 ± 6.5	5.5 ± 2.6	2.4 ± 1.1	> 100	7.7 ± 2.1	4.375 ± 1.25	n.d.	14.25 ± 7.63
**Oxaliplatin**	1.5 ± 0.5	4.7 ± 2.3	4.3 ± 2.5	5.7 ± 4.04	4.7 ± 1.5	2.83 ± 0.15	3.0 ± 0.006	2.5 ± 0.26	4.83 ± 2.25
**PF477736**	1.17 ± 0.35	1.77 ± 0.87	0.027 ± 0.003	0.027 ± 0.004	0.9 ± 0.21	0.23 ± 0.025	0.0825 ± 0.005	0.54 ± 0.37	0.08 ± 0.02
**VE822**	0.68 ± 0.36	1.86 ± 0.9	0.23 ± 0.03	0.58 ± 0.24	1.5 ± 0.5	1.08 ± 0.03	0.725 ± 0.29	1.37 ± 0.25	1.95 ± 0.9
	% dead <30	30<% dead <50		50<% dead <70	% dead >70				
	**Cytostatic**			**Cytotoxic**					

## Data Availability

The data presented in this study are available on request from the corresponding authors.

## Supplementary Materials

The following supporting information can be downloaded at: https://www.mdpi.com/article/10.3390/ijms23105701/s1.
