# Supplementary material for: Multiplexed-Based Assessment of DNA Damage Response to Chemotherapies Using Cell Imaging Cytometry
Source: Int J Mol Sci. 2022 May 20;23(10):5701. doi: 10.3390/ijms23105701 (PMC9145608; doi:10.3390/ijms23105701)
Supplement: Supplementary file 1 [file ijms-23-05701-s001.zip › ijms-1732056-SI.pdf]

Supplementary Material

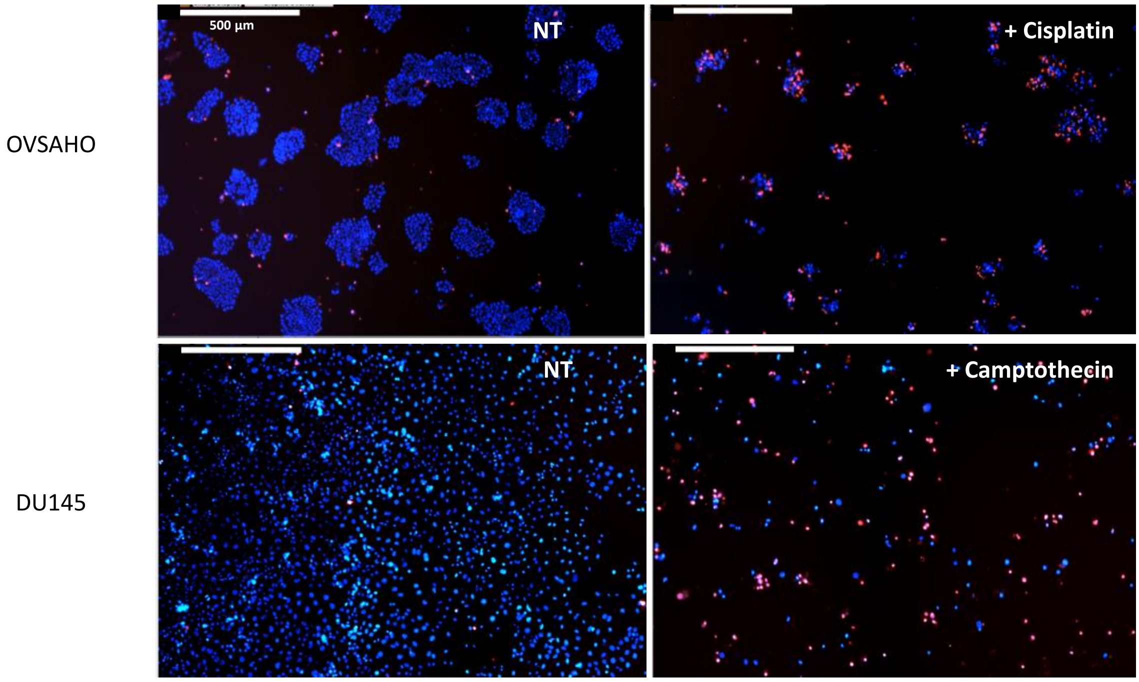

Figure S1. Representative fluorescent images obtained with OVSAHO and DU145 cells. Cells were treated respectively with cisplatin (0.3 µM) and camptothecin (3 µM) for 72 h and stained with propidium iodide (PI) and Hoechst using the Celigo® imaging cytometer.

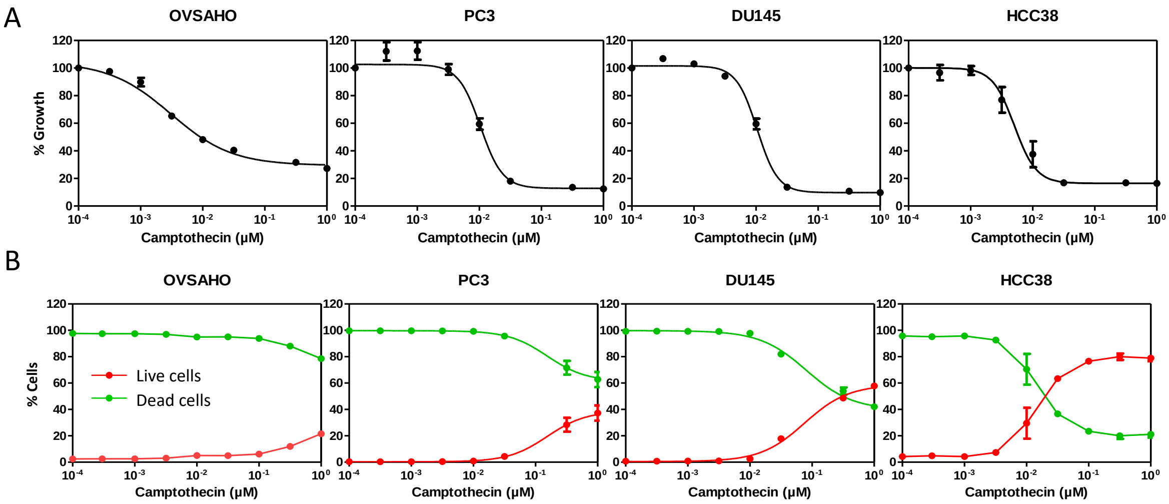

Figure S2. Dual assessment of the cytotoxic and the cytostatic effects of camptothecin in OVSAHO, PC3, DU145 and HCC38 cells using the Celigo® imaging cytometer. Cells were treated with increasing concentration of camptothecin for 72 h and processed as indicated in Figure 1. **(A)** Graphs showing the percentage of growth inhibition as a function of drug concentrations. Total cell number (live + dead cells) in each well was calculated and normalized as compared to untreated controls. Percentages were then plotted as a function of drug concentrations to evaluate IC_{50} values. Results are the mean ± SEM of ≥ 3 independent experiments. **(B)** Plots showing the percentages of dead and alive cells as a function of drug concentrations.

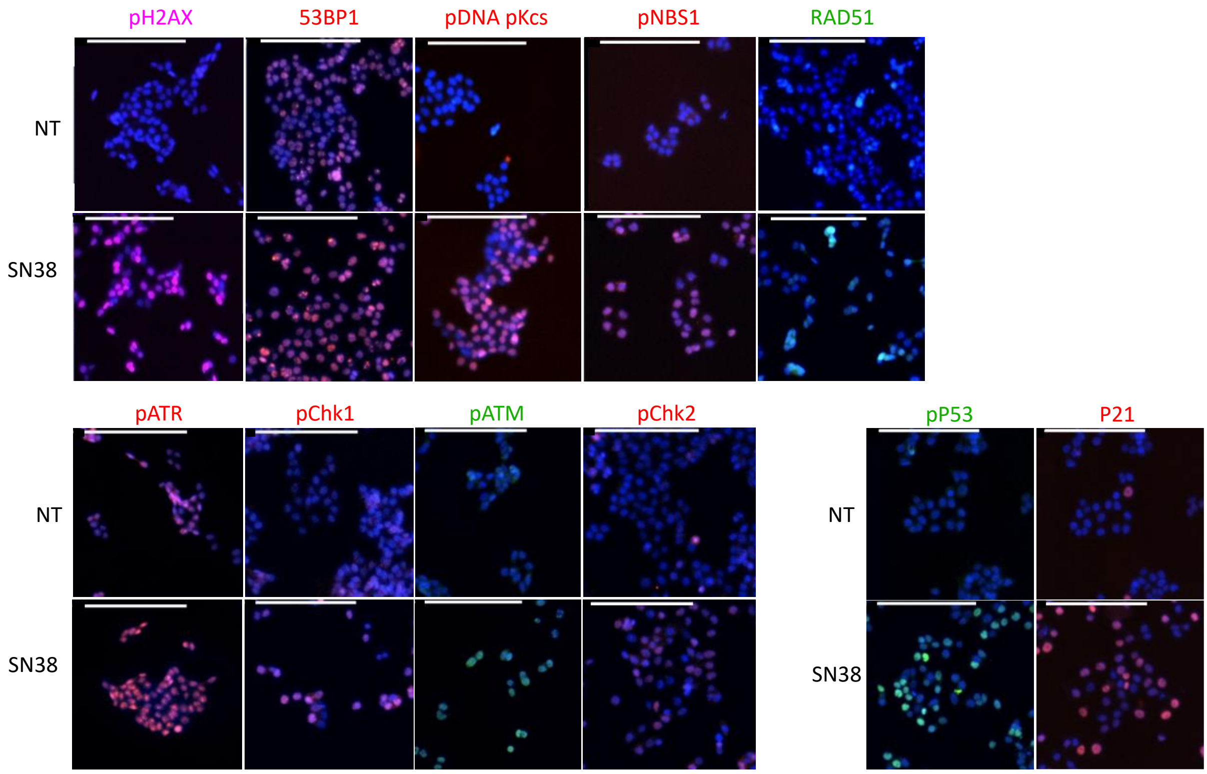

Figure S3. Representative immunofluorescence images that were obtained for each DDR marker using the Celigo® image cytometer. For each marker, several dilutions of primary and secondary antibodies were tested and optimal conditions are summarized in Table 1 and in the Materials and Methods section.

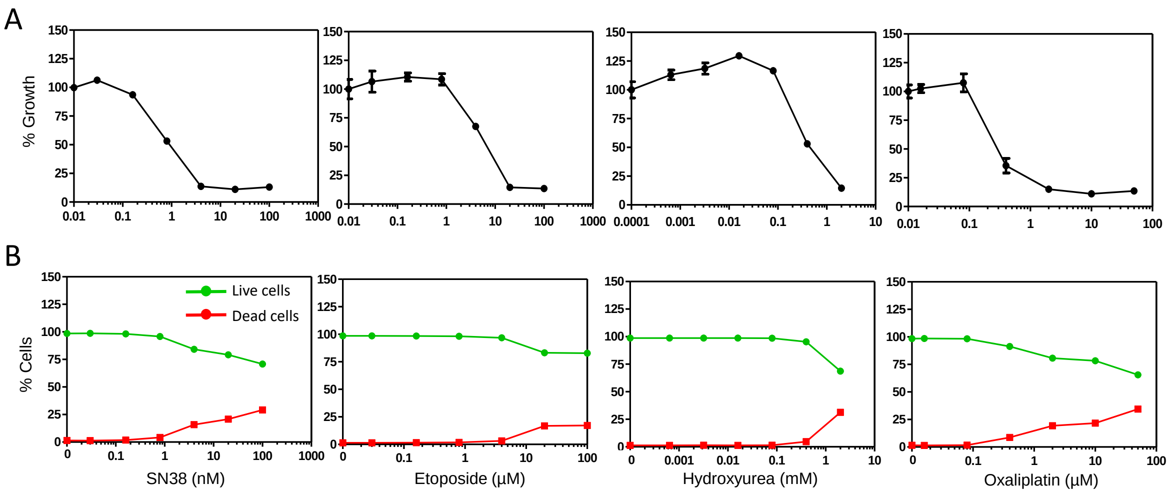

Figure S4. HCT116 cell response to DNA damaging agents**. (A)** Effects of SN38, etoposide, hydroxyurea and oxaliplatin on cell growth. Exponentially growing cells were treated with increasing concentrations of each drug for 72 h and processed as indicated in Figure 1. Percent growth was calculated in comparison with untreated cells and plotted as a function of drug concentrations. **(B)** Dual assessment of cytotoxic and cytostatic effects using the Celigo® imaging cytometer. Cells were treated as in (A) and the percentages of PI-positive dead cells (in red) and calcein-AM-positive live cells (in green) within each well were plotted as a function of drug concentrations. Results are the mean ± SEM of ≥ 3 independent experiments

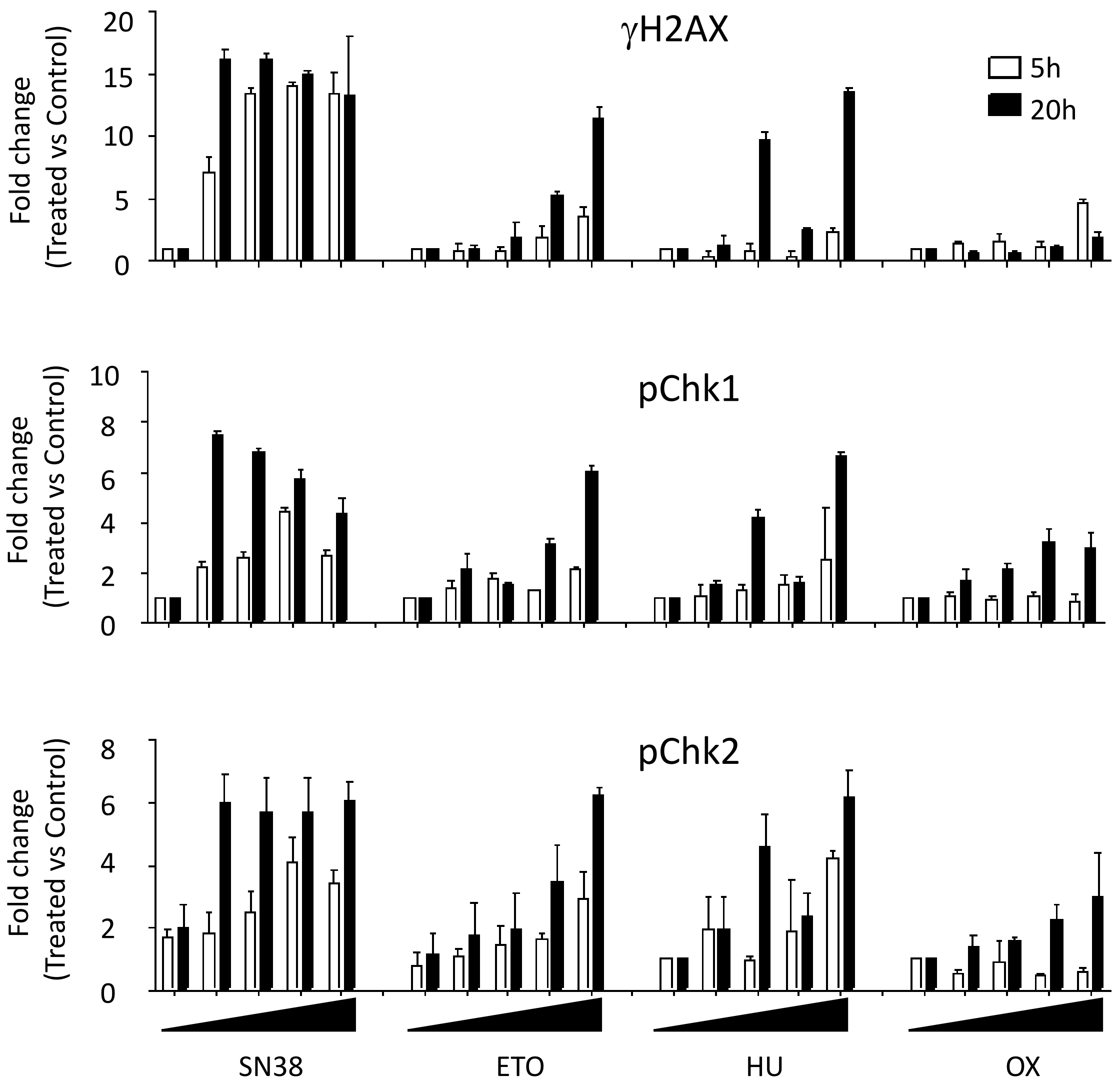

Figure S5. Time-dependent response of HCT116 cells to DNA damage using γH2AX, pCHK1 and pCHK2 as DDR markers. Cells were treated with increasing concentrations of SN38 (0, 0.016, 0.08, 0.4, 2 µM), etoposide (0, 0.8, 4, 20, 100 µM), hydroxyurea (0, 0.016, 0.08, 0.4, 2 mM), or oxaliplatin (0, 0.5, 2.5, 10, 50 µM) for 5 h or 24 h and analyses of fluorescence staining was performed using the Celigo® image cytometer as described in Figure 4. For each DDR marker, the results show the ratios of the average number of fluorescent cells in treated cells as compared to untreated cells. Results are the mean ± SEM of 2-4 independent experiments.

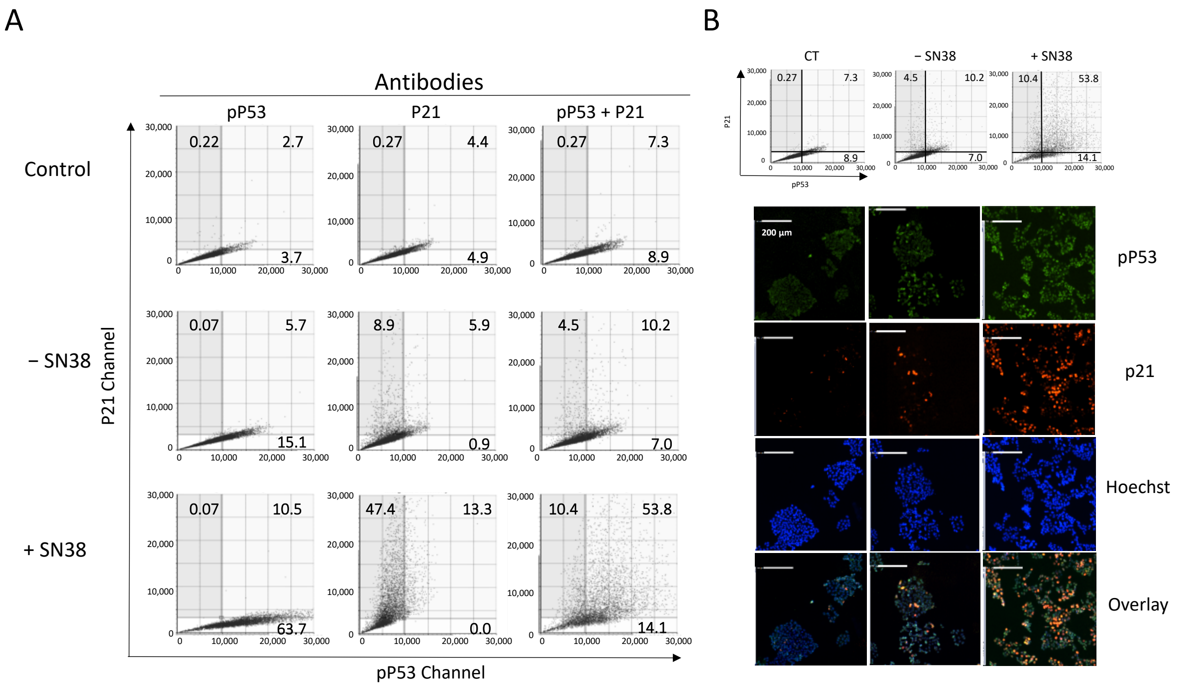

Figure S6. Validation of the multiplexed analyses of pP53 and P21 DDR markers following DNA damage using the Celigo® image cytometer. **(A)** HCT116 cells were treated with SN38 (2 µM) for 24 h and immunofluorescence labelling was performed with either the pP53 antibody alone, the P21 antibody alone, or both antibodies, and analysed using the Celigo® flow cytometry interface using a gating that was determined for the CT condition (untreated cells stained with the secondary antibody alone). Representative dot-plots for one experiment are shown in which percentages of fluorescent positive cells in both channels were determined and indicated in each area of the gating. **(B)** Representative immunofluorescence images that are obtained for pP53 (green) and P21 (red) dual labelling following treatment with SN38. Nuclei are labelled with Hoechst.

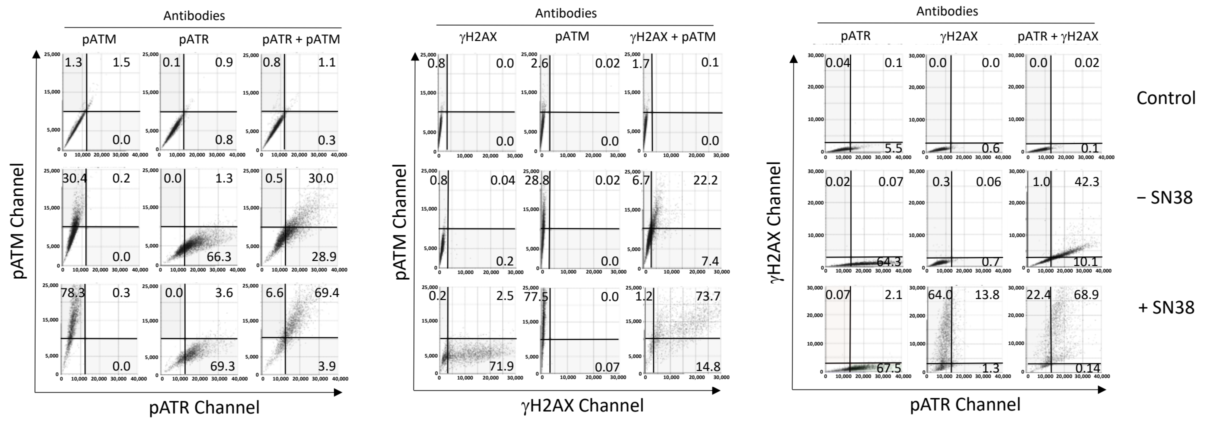

Figure S7. Validation of the multiplexed analyses of pATR, pATM and γH2AX DDR markers following DNA damage using the Celigo® image cytometer. Following treatment with SN38, cells were stained with antibodies alone or with the different combinations of two antibodies and images were analysed using the Celigo® flow cytometry interface as indicated in figure S6. Dot-plots are the results of one representative experiment.

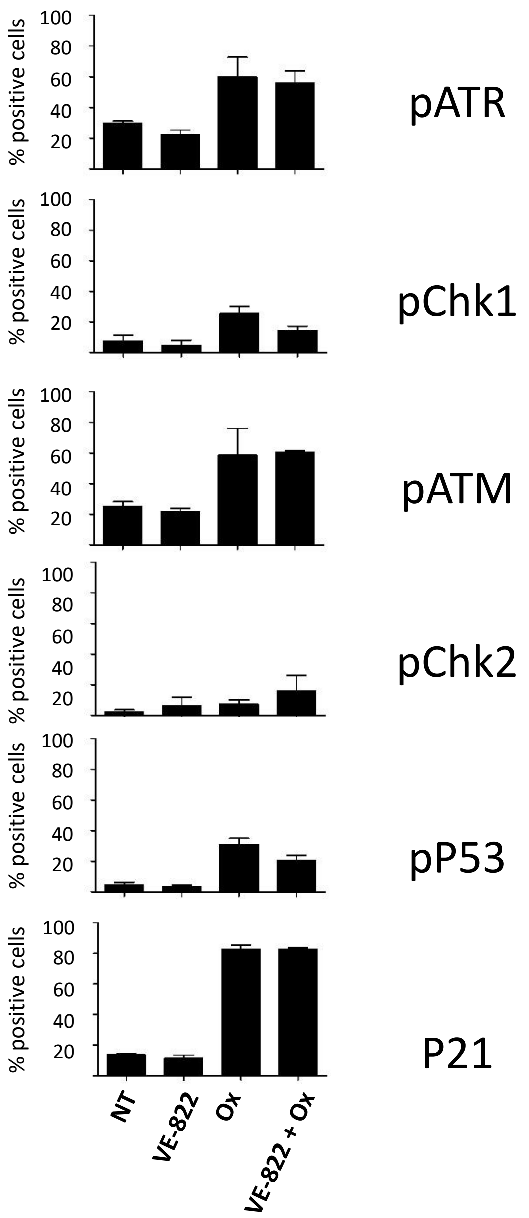

Figure S8. Comparative analyses of DDR markers following treatment with the VOX combination using image cytometry. HCT116 cells were treated with VE-822 (1 µM), oxaliplatin (2.5 µM) or the combination of both for 20 h and protein extracts were performed to analyse the indicated DDR markers using the corresponding antibodies described in Table 1. Quantification of fluorescent positive cells were determined for each DDR marker as described in Materials and Methods. Results are the mean ± sd of triplicates of two independent experiments.

Table S1. List of the primary antibodies used in this study.

Target protein	Company	Reference	Species	Dilutions Celigo®	Dilutions WB
ATM	Cell Signaling Technology	2873	Rabbit	–	1/1000
phospho-ATM (Ser1981)	Cell Signaling Technology	13050	Rabbit	–	1/100
phospho-ATM (Ser1981)	Santa Cruz Biotechnology	sc-47739	Mouse	1/100	–
ATR	Cell Signaling Technology	13934	Rabbit	–	1/1000
phospho-ATR (Thr1989)	Genetex	GTX128145	Rabbit	1/1000	1/500
CDK2	Cell Signaling Technology	2546	Rabbit	–	1/1000
phospho-CDK2 (Thr160)	Cell Signaling Technology	2561	Rabbit	–	1/1000
CHK1	Cell Signaling Technology	2360	Mouse	–	1/1000
phospho-CHK1 (Ser345)	Cell Signaling Technology	2348	Rabbit	1/50	1/1000
CHK2	Cell Signaling Technology	2662	Rabbit	–	1/1000
phospho-CHK2 (Thr68)	Cell Signaling Technology	2661	Rabbit	1/50	1/1000
phospho-DNA-PKcs (Ser2056)	Abcam	ab124918	Rabbit	1/500	1/1000
GAPDH	Cell Signaling Technology	5174	Rabbit	–	1/1000
p21 WAF1/CIP1	Cell Signaling Technology	2947	Rabbit	1/200	1/1000
p53	Santa Cruz Biotechnology	sc-126	Mouse	–	
phospho-p53 (Ser15)	Cell Signaling Technology	9286	Mouse	1/200	1/1000
β-tubulin	SIGMA	T4026	Mouse	–	1/100
phospho-H2AX (ser139)	Abcam	ab195189	Rabbit	1/1000	–
Rad51	Abcam	ab196449	Rabbit	1/100	–
53BP1	Novus Biologicals	nb100-304	Rabbit	1/6000	–
